# Grand Rounds: Could Occupational Exposure to *n*-Hexane and Other Solvents Precipitate Visual Failure in Leber Hereditary Optic Neuropathy?

**DOI:** 10.1289/ehp.9245

**Published:** 2006-09-19

**Authors:** Valerio Carelli, Flavia Franceschini, Silvia Venturi, Piero Barboni, Giacomo Savini, Giuseppe Barbieri, Ettore Pirro, Chiara La Morgia, Maria L. Valentino, Francesca Zanardi, Francesco S. Violante, Stefano Mattioli

**Affiliations:** 1 Dipartimento di Scienze Neurologiche, Alma Mater Studiorum—University of Bologna, Bologna, Italy; 2 Dipartimento di Sanità Pubblica, Azienda USL Bologna, San Giorgio di Piano, Bologna, Italy; 3 U.O. Medicina del Lavoro, Policlinico S.Orsola-Malpighi, Alma Mater Studiorum—University of Bologna, Bologna, Italy; 4 Centro di Oftalmologia Salus, Bologna, Italy

**Keywords:** environmental trigger, Leber hereditary optic neuropathy, mitochondrial DNA, neurotoxin, *n*-hexane, occupational exposure, solvents

## Abstract

**Context:**

Leber hereditary optic neuropathy (LHON) is a maternally inherited loss of central vision related to pathogenic mutations in the mitochondrial genome, which are a necessary but not sufficient condition to develop the disease. Investigation of precipitating environmental/occupational (and additional genetic) factors could be relevant for prevention.

**Case presentation:**

After a 6-month period of occupational exposure to *n*-hexane and other organic solvents, a 27-year-old man (a moderate smoker) developed an optic neuropathy. The patient had a full ophthalmologic and neurologic investigation, including standardized cycloergometer test for serum lactic acid levels and a skeletal muscle biopsy. His exposure history was also detailed, and he underwent genetic testing for LHON mitochondrial DNA mutations. The patient suffered a sequential optic neuropathy with the hallmarks of LHON and tested positive for the homoplasmic 11778G → A/ND4 mutation. Routine laboratory monitoring revealed increased concentrations of urinary 2.5 hexandione (*n*-hexane metabolite) and hippuric acid (toluene metabolite) in the period immediately preceding the visual loss.

**Discussion:**

In a subject carrying an LHON mutation, the strict temporal sequence of prolonged appreciable occupational exposure followed by sudden onset of visual loss must raise a suspicion of causality (with a possible further interaction with tobacco smoke).

**Relevance:**

In this article, we add to the candidate occupational/environmental triggers of LHON and highlight the need for appropriate case–control (and laboratory) studies to validate the causal effect of mixed toxic exposures.

## Case Presentation

In March 2002, a 27-year-old man presented with sudden loss of central vision in the right eye. Visual loss in the left eye followed within 18 days. At the time of onset, the patient was a very light occasional drinker and a moderate smoker (15 cigarettes/day for 5 years; unfortunately, cotinine and *trans*-3′-hydroxycotinine were not measured to assess tobacco smoke exposure). He suffered from chronic headaches, which responded to common painkillers. The headaches had reportedly worsened when he started work in a glue factory in 1998 (he had previously been an electrician) and worsened remarkably when he was transferred to the glue preparation department, where he had worked for 6 months before the loss of vision. At fundus examination, both eyes showed micro-angiopathy and pseudoedema of the optic disc. A cycle of corticosteroids was ineffective at this time. One month later, visual acuity was 1/100 in the right eye and 7/10 in the left eye. We observed the patient in June 2002 and found a bilateral paleness of optic discs and the inability to count fingers with either eye; at that time, the patient complained of disturbing photopsias. A therapy with idebenone (270 mg/day) and brimonidine (2 drops, 3 times/day) was initiated (Mashima et al. 2000; Newman et al. 2005). Mitochondrial (mt) DNA testing on a blood sample was positive for homoplasmic 11778G → A/ND4 mutation. Haplogroup definition showed that this patient’s mtDNA belonged to haplogroup H (DdeI 10394-, AluI 7025-, MseI 14766-) (Torroni et al. 1997). These findings are consistent with a diagnosis of Leber hereditary optic neuropathy (LHON), a maternally inherited loss of central vision that preferentially affects young men (Man et al. 2002); three mtDNA point mutations (positions 11778G → A/ND4, 3460G → A/ND1, and 14484T → C/ND6) are found in over 90% of patients worldwide.

**Figure 1:**
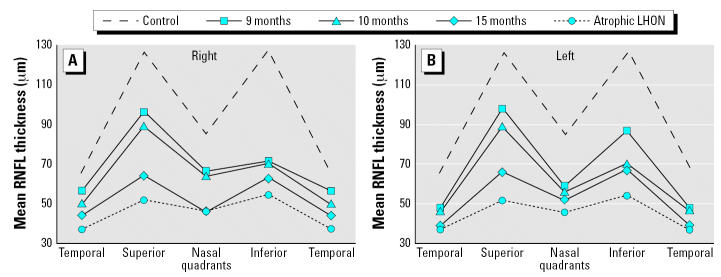
RNFL thickness in each quadrant of the eye, as measured by optical coherence tomography. (*A*) Right eye. (*B*) Left eye. Solid lines refer to the patient’s eyes at 9, 10, or 15 months of follow-up; dashed and dotted lines refer to historical groups of healthy subjects (control) and of patients in an advanced stage of LHON with atrophy of the optic nerve (Barboni et al. 2005).

Until January 2003, visual function progressively deteriorated, reaching almost complete loss of vision (apart from hand motion) with rapid, involuntary eyeball oscillation (nystagmus). Progression of the fiber loss was documented by serial optical coherence tomography (Barboni et al. 2005). Figure 1 shows the retinal nerve fiber layer (RNFL) thickness in each quadrant, as measured by optical coherence tomography, for each of the patient’s eyes after 9, 10, and 15 months, alongside values for historical groups of healthy subjects and of patients in an advanced stage of LHON (with atrophy of the optic nerve) (Barboni et al. 2005). Compared with the control group, both of the patient’s eyes at 9, 10, and 15 months follow-up showed a progressively thinner RNFL in all quadrants.

By January 2003 (10 month follow-up), the patient needed assistance to walk. After June 2003, he reported a slow but progressive improvement. A full clinical reevaluation was performed in September 2005; abnormal findings were limited to poor visual acuity, pale optic discs, and sluggish pupillary light reaction. On a standardized cycloergometer test (Montagna et al. 1995), serum lactic acid levels were almost normal (11.5, 11.9, 23.0, and 13.0 mg/dL; normal range, 5.8–22.0 mg/dL). Folate and vitamin B_12_ were normal, whereas creatine phosphokinase was high (289 U/L; normal value, < 170 U/L). Muscle biopsy showed nonspecific changes, with an observable parcellar subsarcolemmal increase of succinic dehydrogenase staining, indicating some mitochondrial proliferation. The patient is now able to count fingers and is without nystagmus (both eyes). He is able to walk without assistance and is continuing the same treatment.

### Maternal lineage reconstruction

**Figure 2:**
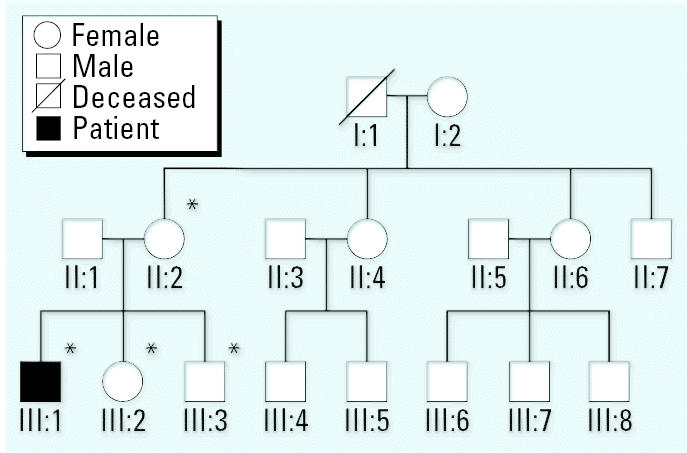
Three-generation reconstruction of the patient’s maternal line. mtDNA analysis of the patient and his mother and siblings (asterisks) revealed the homoplasmic 11778/ND4 LHON mutation in all four of them; however, the patient was the only family member affected.

Because the mutations associated with LHON affect complex I subunits and, in most families, are homoplasmic (100% of mtDNA copies are mutant), we performed genealogical reconstruction for the maternal lineage of the patient’s family. After obtaining informed consent, we collected blood from the patient’s siblings and mother for mtDNA testing by restriction fragment length polymorphism analysis, as described by Torroni et al. (1997). These subjects were unaffected carriers of the homoplasmic 11778/ND4 LHON mutation. No history of occupational or environmental exposure to solvents (or other particular toxic substances) was reported for any of the family members tested (Figure 2). These results are in accordance with the current concepts regarding LHON; the mtDNA mutation is a necessary but not sufficient condition for LHON, and only a minority of carriers develops optic neuropathy. Penetrance is incomplete (and lower in women), implying that additional mitochondrial/nuclear genetic factors and possibly also environmental factors contribute to phenotypic expression of LHON (Carelli et al. 2003); however, these triggering factors are poorly defined.

### Occupational exposure history

Between 1998 and 2000 the patient had worked in the “hot-melt” department, where he controlled hot layering of a synthetic adhesive containing styrene-isoprene copolymer, isopropilic alcohol, toluene, xylene, and octanes. An environmental exposure assessment requested by factory inspectors in 2003 indicated vapor levels of the individual solvents were 10–20% of American Conference of Governmental Industrial Hygienists (ACGIH) threshold limit values (TLVs) (ACGIH 2005). During 1998–2000, the patient recalled experiencing nausea, dizziness, asthenia, and paresthesia of the upper and lower limbs (in addition to chronic headaches) whenever he had to clean the machines—a task he performed using a mixture of styrene, isopropilic alcohol, toluene, xylene, and octanes. In 2000–2001 the patient worked in the rubber*-*mincing department, where he was indirectly exposed to *n*-hexane and toluene (no quantitative data are available). In the glues preparation department, where the patient worked from October 2001 to March 2002, exposure to *n*-hexane and toluene was presumably more direct because of the nature of his duties: He prepared coloring mixtures containing rubber, hydrocarbonic resin of toluene, and *n*-hexane in a closed production cycle, and he opened dissolver machines 5 times/day to determine if the tanks were empty. The only available environmental monitoring data for the department (again requested post hoc in 2003) report peak values of 10.7 mg/m^3^ for *n*-hexane (ACGIH TLV, 176 mg/m^3^) and 1.5 mg/m^3^ for toluene (ACGIH TLV, 188 mg/m^3^). In this department, the patient reported constantly feeling nauseous and dizzy. Also, during the 6-month period, four overflow accidents occurred, each reportedly followed by several hours of intensified dizziness and nausea, asthenia, tingling of the upper and lower limbs, and dysesthesia of the hands (the same set of symptoms the patient recalled experiencing after using solvents for cleaning tasks in the hot-melt department). Biological monitoring data routinely collected throughout the period of employment indicated that after the patient moved to the glue preparation department, he had a > 4-fold increase in urinary 2,5-hexandione (a neurotoxic metabolite of *n*-hexane) from 0.5–0.9 to 4.1 mg/L, accompanied by a > 1.5-fold increase in urinary hippuric acid (a toluene metabolite) from 360–700 to 1,040 mg/L (roughly corresponding to an increase from 0.26–0.5 to 0.74 g/g creatinine).

## Discussion

In a subject without specific genetic predisposition, the appreciable biological exposure levels of urinary 2,5-hexandione (4.1 mg/L) and hippuric acid (1,040 mg/L, roughly equivalent to 0.74 g/g creatinine) encountered in the glue factory would not elicit concern for major neurologic involvement [although the biological exposure indices adopted by the ACGIH are 0.4 mg/L for 2,5-hexandione and 1.6 g/g creatinine for hippuric acid (ACGIH 2005)]. The observed association could have been casual because the patient was a smoker and the presentation of LHON was rather typical. Nevertheless, in a subject carrying an LHON mutation, the strict temporal sequence of a prolonged appreciable occupational exposure followed by sudden onset of visual loss must raise a suspicion of causality.

At present, no specific evidence is available for any association between solvents and LHON. Johns et al. (1992) reported an isolated case of an LHON patient with occupational exposure to solvent fumes—a firefighter repeatedly exposed to xylene, toluene, and methylethylketone, as well as smoke and other fumes. It is reasonable to suppose that both in the case of this firefighter and in our patient, it may have been an acute exposure to solvents that triggered the onset of LHON. Recently, a late-onset LHON case was reported to follow a long history of occupational exposure to polycyclic aromatic hydrocarbons (Rufa et al. 2005). Putative associations have been suggested between solvents and other optical/ retinal neuropathies (Maruff et al. 1998; Meadows and Verghese 1996). In a report on 15 workers exposed to *n-*hexane in an adhesive bandage factory, 11 workers showed macular changes and 1 had central retinopathy (Raitta et al. 1978). Several case reports describe optic neuropathy and/or hearing loss among glue sniffers (Ehyai and Fremon 1983; Ogawa et al. 1988; Williams 1988). Furthermore, peripheral neuropathy and sensorineural hearing loss was described in a painter exposed to a mixture of organic solvents, including toluene and xylene (Moshe et al. 2002). Occupational exposure to (or sniffing of) toluene is thought to have an acute effect on color vision that is capable of inducing retinal and optic nerve degeneration (Gobba and Cavalleri 2003; Kiyokawa et al. 1999). Interestingly, *in vitro* data suggest that the neurotoxic effects of styrene are related to mitochondrial damage, in addition to oxidative stress (Darè et al. 2004). Regarding isopropanol, experiments on rats showed that inhalation of this alcohol boosts renal and hepatic microsomial metabolism of *n*-hexane, leading to increased formation of neurotoxic metabolites (Zahlsen et al. 1984). Animal studies on *n*-hexane exposure also indicate that 2,5-hexanedione [the known neurotoxic *n*-hexane metabolite (Spencer and Schaumburg 1985)] could provoke premature or accelerated deterioration in vision (Spencer and Schaumburg 1978) and, by reducing the rate of ATP synthesis, decrease the endogenous concentration of ATP in brain mitochondria (Sickles et al. 1990). Moreover, *n*-hexane uncouples mitochondrial respiration by a nonprotonophoric mechanism (Canton et al. 1996). The uncoupling effect and impaired ATP synthesis induced by *n*-hexane (or its neurotoxic metabolite) suggest that this compound could be a candidate trigger for LHON in our patient. Although *n*-hexane and toluene toxicity is not thought to be severe at our patient’s exposure level, the possible neurotoxic potentiation of the mixed exposure has to be considered (Noraberg and Arlien-Soborg 2000). Solvents, their metabolites, and tobacco smoke derivatives (the patient was a moderate smoker) could interact with the biochemical defect related to the LHON mutation on complex I (Carelli et al. 2004). Although it is not possible to draw conclusions about any triggering role of *n*-hexane, toluene, and/or other solvents and smoking exposures, it is noteworthy that the patient did suffer other signs and symptoms suggestive of toxic exposure before the onset of LHON.

Many aspects of the complex etiology of LHON remain poorly defined (Man et al. 2002; Carelli et al. 2004). The incomplete penetrance and the male propensity clearly indicate that the mtDNA mutation is not the only determinant for disease expression. Research is required into secondary factors modulating the clinical expression of LHON, which could include toxic exposures, alcohol/tobacco abuse, and metabolic dysfunctions (Lachmund and Mojon 2006). One case–control study failed to reveal associations with smoking and/or alcohol drinking (Kerrison et al. 2000), whereas another reported associations with smoking and aspecific occupational toxic exposures (Sadun et al. 2003). Nevertheless, individuals exposed to substances with neurotoxic properties (including ethanol and smoke) affecting hepatic or renal function or solvent metabolism generally tend to be particularly vulnerable to exposure to organic solvents (Spencer and Schaumburg 1985).

## Conclusion

Epidemiologic and biological studies are needed to explore secondary factors modulating the clinical expression of LHON, including metabolic, lifestyle, environmental, and occupational triggers. Possible differential effects of chronic and acute exposures to solvents and other toxic substances require consideration.
